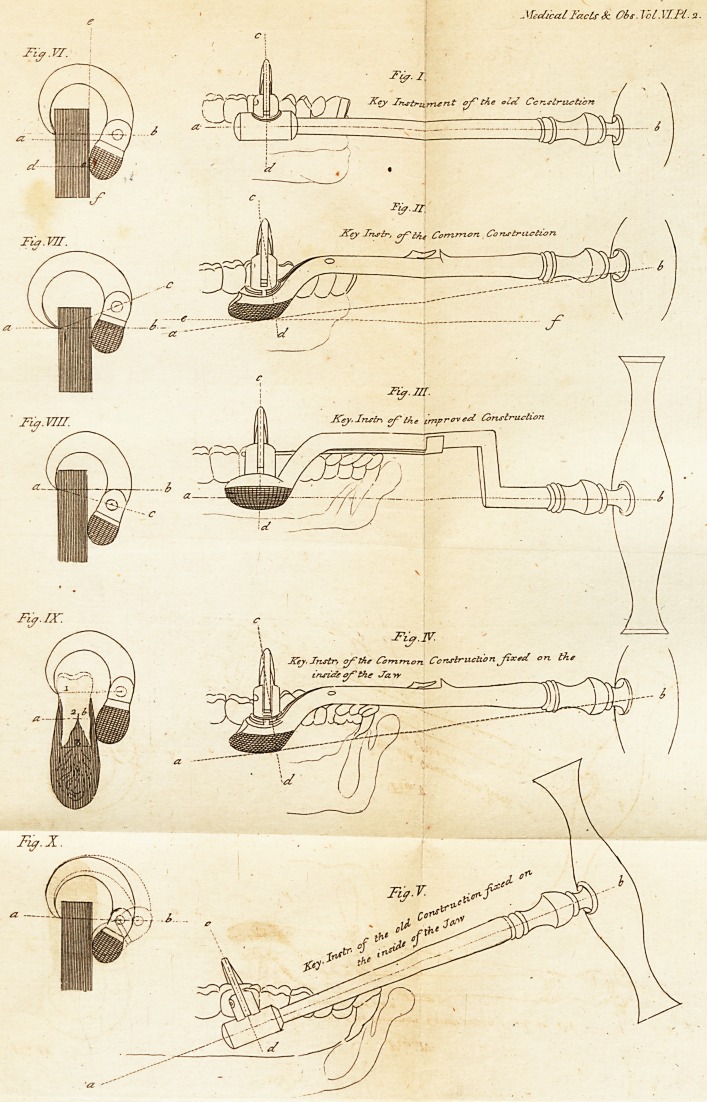# An Account of a Key Instrument of a New Construction; with Observations on the Principles on Which It Acts, in the Extraction of Teeth, and on the Mode of Applying It

**Published:** 1795

**Authors:** Robert Clarke

**Affiliations:** Surgeon at Sunderland, in the County of Durham.


VIII. *An Account of a Key Inſtrument of a new Conſtruction; with Obſervations on the Principles on which it acts, in the Extraction of Teeth, and on the Mode of applying it. By Mr.* Robert Clarke, *Surgeon at Sunderland, in the County of Durham. Communicated in a Letter to Mr.* Anthony Carliſle, *Surgeon of the Weſtminſter Hoſpital, and Reader of Anatomy in London; and by him to Dr.* Simmons.

*To Mr.* CARLISLE.

SIR,

WITH this I ſend you a Key Inſtrument, for the Extraction of Teeth, of a conſtruction different from any in common uſe, and which in practice fully anſwers to the expectations I had formed, *a priori*, from a careful examination of the principles of its action.

I cannot, perhaps, give you a clearer idea of its advantages, than that which you will obtain by purſuing the ſame train of inveſtigation which I followed myſelf. I ſhall therefore proceed to lay it before you, that I may more thoroughly convince you of the propriety of the alteration I have made, or be corrected by your pointing out any error I may have fallen into.

In

Fig. I.
Key Instrument of the old Construction
Fig. II.
Key Instr. of the Common Construction
Fig. III.
Key. Instr. of the improved Construction
Fig. IV.
Key. Instr. of the Common Construction fixed on the inside of the Jaw
Fig. V.
Key. Instr. of the old Construction fixed on the inside of the Jaw
Fig. VI.
Fig. VII.
Fig. VIII.
Fig. IX.
Fig. X.

In the firſt place then, it appeared to me that as the fulcrum, or point, upon which the tooth is carried round as on a center, is that part of the bolſter which reſts upon the gums, the axis of motion of the inſtrument would always be found by drawing a line through that point and the middle of the handle; and conſequently that the old conſtruction of the Key Inſtrument was free from an inconvenience which attends the more modern one: I mean the axis of the bolſter and axis of the ſhank making an angle with each other; on which account it is diſpoſed to ſhift its point of action on the gums, and to raiſe the tooth in a plane inclined to the throat, inſtead of a vertical one, as may be clearly ſeen by inſpecting Figures I. II. (Plate II.*) where *a*, *b*, repreſent the axis of motion; *c*, *d*, the direction in which each inſtrument raiſes the tooth; and *e*. *f*. (Fig. II.) the axis of the bolſter.

Now as the line of direction in Fig. I. is perpendicular to the jaw, it is needleſs to ſay that it is highly preferable to Fig. II. where the line of direction is inclined backward, making the

* It ſeems right to obſerve here, that all the figures of this plate are on a reduced ſcale of two thirds of their proper ſize.

extraction extraction of the tooth more difficult, and exposing that which is situated behind it to be driven from its socket, or even to be caught in the arch of the claw. Besides this, the bolster rests only upon the corner *d*, adding greatly to the injury of the gums.

The construction then of the Key-instrument delineated in Fig. I. would seem perfect, were it not that in drawing teeth inwards, with respect to the jaw, the fore teeth prevent its due application, confining it to the direction represented in Figure V.

To remedy this imperfection I have made the instrument with a bend in its shank, to clear the fore teeth, and to allow its proper application, as in Figure III. where the same observations and references apply as in Figure I. and therefore it is unnecessary to repeat them. But in order that the comparative merits of the three instruments may be seen at a glance, I have added Figures IV. and V. wherein the axis of motion, and the direction of the rising tooth, are shown by dotted lines.

Having fully considered what relates to the direction of the tooth, I shall next examine the mechanism which takes hold of it. For this purpose recourse must be had to the engraving.

Let

Let *a*, *b*, *c*, Figure VI. reprefent an end view of a Key inftrument, fixed upon a piece of hard, fmooth wood. Then it is obvious, that if it be turned from left to right, by means of its handle, it will break the wood in the direction *d*, *c*, and caufe the upper fragment to revolve on the point *c*, as a center. It is equally obvious, that if a line be drawn from the point *a*, croffing the oppofite furface of the folid *e*, *f*, at right angles, the counterpoife of the claw will fall into that line before it can take hold; for then the point *b*, is at the greateft poffible diftance from the furface *e*, *f*; confequently if the inftrument be placed as in Figure VII. the point *c* will defcend; or, if as in Figure VIII. it will afcend until it coincides with the line *a*, *b*.

I fhall now endeavour to apply this to practice. Let 1, 2, 3, in Figure IX. reprefent a tooth with its roots fixed in a fection of the jaw, and its corona engaged in a Key-inftrument; then it will readily appear that upon the action of the inftrument, the tooth will be drawn from its focket, and carried round the point *b*, as a center, rather than the joint fubftance of the tooth and jaw be broken in the line *a*, *b*, as happens in Figure VI. This however happens

 only only under particular circumftances : For if the bolfter be placed too high, the tooth will be broken ; and if too low, the alveolar procefs will always be torn away with it. It is therefore a matter of importance to determine the beft point of contact for the bolfter, and this I have uniformly found to be at two-thirds the depth of the tooth, the claw being fixed at one third, as reprefented in Figure IX.

It will always be eafy to afcertain this point, by attending to the fize of the corona, and the part of the jaw where the tooth is fituated ; and equally fo to make the inftrument act upon it, by ufing a larger or fmaller claw as the cafe may require. For illuftration, however, I fhall refer to Figure X. which reprefents a piece of wood grafped by the tooth inftrument in the fame manner as in Figure VI. Now if a larger claw, fhewn by the dotted line, be ufed, the bolfter will fix higher upon the wood than before. For as the center pin of the claw will always reft in the line *a*, *b*, the bolfter muft rife higher before it can come into contact. But notwithftanding the ufe of a larger or fmaller claw, in proportion to the fize of the tooth, enables us to fix it at a proper height, the ufe of a very difproportionate one is always inconvenient, inconvenient, by depriving us of the ufe of the crank, in drawing teeth inwards, and by encroaching upon the cheeks in drawing them outwards. I have therefore in the conftruction of this inftrument, taken care to make the bolfter of fuch a depth, as to be free from either inconvenience.

The form of the bolfter is by no means a matter of indifference; for if it be too fmall, it prefents fo fmall a furface to the gums, that the preffure made upon them, by the extraction of a tooth moderately firm, cuts them through, and even penetrates the bone, efpecially if the bolfter be of the ufual form. I have therefore been careful to make it of a proper fize, and to give it a prolate fpheroidal figure, as being the leaft difpofed to injure the gums, and applicable with exactnefs and eafe to all parts of the mouth; and in order ftill further to guard againft this bruifing of the gums, I wrap the bolfter to the thicknefs of a line, with tow, wound on as tight as I can, before I flide forward the bolt and put in the claw.

I have alfo been attentive to the form of the claws, that they may touch the tooth only with their points. And the inftrument is fo contrived, that they can be quickly changed or turned turned to an oppoſite direction as the caſe may require: this is done by means of a ſliding bolt, inſtead of a ſcrew, which paſſes through the claws.

I have always found that when the tooth is to be turned from right to left in drawing it, that the handle anſwers beſt placed perpendicularly; and when from left to right, horizontally. The reaſon of this will be obvious, if we conſider that in the firſt caſe, the pronator muſcles of the operator's arm, which are thoſe exerting the force, act with moſt advantage when the hand is vertical; and in the ſecond caſe, that the ſupinators act moſt advantageouſly with the hand prone. I have therefore contrived the handle ſo that it may be eaſily turned, as often as there is occaſion to turn the claw.

I am, Sir, &c.

*Sunderland*, ROBERT CLARKE.
Aug. 18, 1794.

IX. *An*

## Figures and Tables

**Fig. I. Fig. II. Fig. III. Fig. IV. Fig. V. Fig. VI. Fig. VII. Fig. VIII. Fig. IX. Fig. X. f1:**